# Engaging primary care practitioners in quality improvement: making explicit the program theory of an interprofessional education intervention

**DOI:** 10.1186/1472-6963-13-106

**Published:** 2013-03-20

**Authors:** Brigitte Vachon, Bruno Désorcy, Michel Camirand, Jean Rodrigue, Louise Quesnel, Claude Guimond, Martin Labelle, Johanne Fournier, Jeremy Grimshaw

**Affiliations:** 1 School of Rehabilitation, Faculty of Medicine, Université de Montréal, 7077 Park Avenue, Montreal, Quebec H3N 1X7, Canada; 2 Agence de la santé et des services sociaux de la Montérégie, 1255 Beauregard St, Longueuil, Quebec J4K 2M3, Canada; 3 Centre de santé Sutton, 33 Principale St South, Sutton, Quebec J0E 2K0, Canada; 4 Direction générale des services de santé et médecine universitaire, Ministère de la Santé et des Services sociaux, Gouvernement du Québec, Longueuil, Quebec, Canada; 5 Département régional de médecine général, 1255 Beauregard St, Longueuil, Quebec J4K 2M3, Canada; 6 Fédération des médecins omnipraticiens du Québec, 1440 Sainte-Catherine St West, Suite 1000, Montreal, Quebec H3G 1R8, Canada; 7 Centre for Practice-Changing Research, Ottawa Hospital Research Institute, The Ottawa Hospital General Campus, 501 Smyth Road, Box 711, Ottawa, Ontario K1H 8L6, Canada

**Keywords:** Interprofessional continuing education, Quality improvement, Primary care practice, Program theory-driven evaluation

## Abstract

**Background:**

The scientific literature continues to advocate interprofessional collaboration (IPC) as a key component of primary care. It is recommended that primary care groups be created and configured to meet the healthcare needs of the patient population, as defined by patient demographics and other data analyses related to the health of the population being served. It is further recommended that the improvement of primary care services be supported by the delivery of feedback and performance measurements. This paper describes the theory underlying an interprofessional educational intervention developed in Quebec’s Montérégie region (Canada) for the purpose of improving chronic disease management in primary care. The objectives of this study were to explain explicitly the theory underlying this intervention, to describe its components in detail and to assess the intervention’s feasibility and acceptability.

**Method:**

A program impact theory-driven evaluation approach was used. Multiple sources of information were examined to make explicit the theory underlying the education intervention: 1) a literature review and a review of documents describing the program’s development; 2) regular attendance at the project’s committee meetings; 3) direct observation of the workshops; 4) interviews of workshop participants; and 5) focus groups with workshop facilitators. Qualitative data collected were analysed using thematic analysis.

**Results:**

The theoretical basis of the interprofessional education intervention was found to be work motivation theory and reflective learning. Five themes describing the workshop objectives emerged from the qualitative analysis of the interviews conducted with the workshop participants. These five themes were the importance of: 1) adopting a regional perspective, 2) reflecting, 3) recognizing gaps between practice and guidelines, 4) collaborating, and 5) identifying possible practice improvements. The team experienced few challenges implementing the intervention. However, the workshop’s acceptability was found to be very good.

**Conclusion:**

Our observation of the workshop sessions and the interviews conducted with the participants confirmed that the objectives of the education intervention indeed targeted the improvement of interprofessional collaboration and quality of care. However, it is clear that a three-hour workshop alone cannot lead to major changes in practice. Long-term interventions are needed to support this complex change process.

## Background

In Canada, 16 million people or 50.6% of the Canadian population [1] live with a chronic disease [2]. It has been acknowledged that managing these diseases is a complex process and that both the economic and social burdens are significant [3]. For example, at the beginning of this millennium, the Public Health Agency of Canada estimated the direct and indirect costs of cardiovascular diseases at $18.5 billion a year [4], which is about 1.5% of the 2012 GDP [5]. Patient numbers are growing rapidly due to the aging of the population and the greater longevity of people with chronic conditions [6]. In addition, many studies have shown a major gap between usual and best practices in the screening, diagnosis, and treatment of chronic diseases [7]. The challenge faced by practitioners and their organizations in developing and maintaining evidence-based practices is enormous.

Knowledge translation and interprofessional collaboration are two important components of continuous quality improvement of healthcare services. According to the Chronic Care Model [7,8], services delivered to patients should be coordinated and based on the best available evidence. However, this requires a paradigm shift in the way we think about medical care: instead of focusing on the acute needs of individual patients, the Chronic Care Model calls for a thoughtful, organized, proactive approach to improving the healthcare of a patient population [6]. A report issued by Quebec’s public health agency (the Institut national de santé publique du Québec, or INSPQ), based on consultation with experts and a literature review, described the main barriers to implementation of integrated primary healthcare in this province: lack of a clinical-practices information system that could be used to support clinical decision making, remuneration of healthcare professionals in a way that promotes *ad hoc* services rather than the actions required for chronic disease management, lack of organization and delivery of multidisciplinary services in primary care, and lack of an evaluation and feedback culture to facilitate the continuous quality improvement of these services [9].

The scientific literature continues to advocate interprofessional collaboration (IPC) as a key component of primary care. According to D'Amour and collaborators, collaboration is composed of two key elements: the construction of a collective action that addresses the complexity of client needs, and the construction of a team dynamic that integrates each professional’s perspective and in which team members respect and trust each other [10]. Based on a recent Cochrane review, practice-based interventions aimed at increasing interprofessional collaboration through practice changes can improve healthcare and patient outcomes, and have generally proved to be less costly for front-line services [11]. However, high-quality evidence from multi-method studies is still lacking. The College of Family Physicians of Canada (CFCP) has identified some important success factors for interdisciplinary collaboration in primary healthcare, the most important one being investing time in intra-group and inter-disciplinary communication [12]. Processes such as team meetings and current practice audits may also influence interprofessional teamwork. Population and specific-patient needs’ assessments should be key determinants in deciding what kinds of teams are required and how to define interdisciplinary collaboration [12]. The CFCP recommended that primary care groups be created and configured to meet the healthcare needs of the patient population, as defined by patient demographics and other data analyses related to the health of the population being served.

It further recommended that improvement of primary care services be supported by the delivery of feedback and performance measurements [13]. Although providing feedback can be effective, its effects are generally small to moderate. In a recent systematic Cochrane review [14], the effect of using audits and feedback was found to vary widely across the included studies, and the quality of the evidence was moderate. The recommendations made following this extensive review were that feedback may be most effective when it reports greater gaps in health professionals’ performance, is provided more than once (both verbally and in writing), and includes targets and an action plan.

In 2008, the regional department of general medicine in Quebec’s Montérégie region (Département régional de médecine général, or DRMG) in collaboration with the Montérégie health and social services agency (Agence de la santé et des services sociaux de la Montérégie, or ASSSM) and the Quebec federation of general practitioners (Fédération des médecins omnipraticiens du Québec, or FMOQ), launched COMPAS, the collective for best practices and improvement in healthcare and services in family practice in Montérégie (*Collectif pour des meilleures pratiques et l’amélioration des soins et services en médicine de famille*). The objective of this ongoing project is to engage front-line healthcare professionals in a process of continuously improving the services they offer to persons suffering from chronic diseases in their territory. Interprofessional learning workshops are offered to physicians, nurses, pharmacists, and other professionals from the same region. In these workshops, information taken from clinical-administrative databases in the healthcare system are presented to the participants to provide them with feedback on the community of patients they are treating and on their practices, and to engage them in the process of working together to develop a collaborative quality-improvement project. Diabetes was identified by the project steering committee as the first priority target.

In 2010, researchers joined the COMPAS project team to support the development, implementation, and evaluation of this quality improvement strategy. Since continuing education workshops are a complex intervention composed of several interacting components [15,16], the research team proposed that the project leaders begin by developing the theory underlying their interprofessional educational intervention. One of the main challenges involved in the innovative COMPAS approach is that of clearly identifying the intervention’s most important and measurable outcomes and also of explaining explicitly how it is intended to achieve change and the expected outcomes. As recommended by the UK Medical Research Council [15] with regard to the development and evaluation of complex interventions, a vitally important early task is to develop a theoretical understanding of the likely process of change by drawing on existing evidence and theory, supplemented if necessary by new primary research, such as interviews with those targeted by the intervention or involved in its development or delivery.

### Aim of the study

The aim of our study was to describe the theory underlying the interprofessional educational intervention developed by the COMPAS project. The objectives were 1) to explain explicitly the set of assumptions held about the manner in which the program relates to its expected outcomes; 2) to describe the components of the complex intervention in detail; 3) to assess the intervention’s feasibility and acceptability, as well as possible improvements to it, and 4) to describe the intervention’s preliminary impacts. This paper will focus on the results obtained with respect to objectives one to three.

## Methods

A program impact theory-driven evaluation approach was used [17,18]. This approach allows for the evaluation of complex interventions implemented in a community setting where researchers have little control over characteristics of the participants, program, and organizational contexts. Program impact theory-driven evaluation is participatory; it emphasizes the importance of working collaboratively with relevant stakeholders from the outset to develop both a common understanding of the program and realistic expectations by tailoring the evaluation to meet the agreed-on values and goals. A program impact theory is a conceptual framework describing how a program intends to work to affect outcomes and the conditions under which these processes are believed to operate. It explicitly details the cause-and-effect sequences that link the intervention components to outcomes [17]. The study protocol and consent form of this study were previously approved by the Ethics Committee of the Ottawa Hospital Research Institute.

Multiple sources of information were used to develop the program impact theory [17] underlying the COMPAS project: 1) a literature review and a review of documents describing the program’s development (minutes of the coordination, steering, and pedagogical committees’ meetings; previous presentations given on the project’s goals and development); 2) regular attendance at the project’s committee meetings; 3) direct observation of the workshops; 4) interviews of workshop participants; and 5) focus groups with workshop facilitators.

When the research team joined the project team (BV, BD, JG), its first task was to familiarize itself with the project. The members reviewed all documentation produced since the beginning of the initiative. At that time, it had already been decided that the intervention would take the form of an interprofessional workshop, but the content and specific learning strategies to be used were not yet defined. One of the principal investigators (BV) joined the project’s various committees to gain a more in-depth understanding of the intervention and also contribute to its development. Preliminary versions of the program impact theory were developed and validated with the project leaders. Six workshops were observed directly to assess the plausibility of the program impact theory. A member of the research team (BV) attended these six sessions and took notes on the level of participation, acceptability of the intervention, characteristics of the educational process, group dynamics, level of interprofessional exchange, facilitation process, time constraints, and the workshop’s immediate impact. The level of satisfaction with the workshop was also documented using the FMOQ’S standard CME evaluation form.

Convenience sampling was used to recruit participants, attending different workshop sessions and from different disciplines (physicians, nurses, pharmacists), that would help the project team understand and validate the underlying program impact theory and the outcomes produced by the intervention. At the end of each workshop, all participants were approached to participate in an individual 30 minutes telephone interview. Because of work overload, it was difficult to recruit primary healthcare professionals willing to take the time to participate in these interviews. However, ten individuals volunteered to be called four to eight weeks after the workshop session and nine were finally interviewed (1 physician, 2 pharmacists and 6 nurses). All participants interviewed signed a consent form beforehand. An interview guide (see Table 1 for the questions asked) was used to conduct the semi-structured interviews. The interviews were conducted by the project coordinator (BD), lasted between 15 minutes and one hour, and were recorded on a digital audio-recorder. In order to get familiarized with the data, the project coordinator listened to the interview at least twice and made a summary of the content of each interview. The principal investigator (BV) reviewed all the summaries while listening to each interview. Using thematic analysis [19], each summary was initially coded and categories were created to document the workshop’s objectives and impact as perceived by the interviewees. The preliminary results were discussed by two members of the research team (BV and BD), and themes were extracted by means of constant comparative analysis: comparing the different participants’ answers in order to conceptualize their perspectives and how they related to the previously developed program impact theory.

**Table 1:** Interview guide used with workshop participants

–	According to you, what were the objectives of this workshop?
–	What did you learn during this workshop?
–	Do you plan to make or have you already made any changes to your practices?
–	Did the workshop allow you to identify these changes?
–	What do you think of the pedagogical strategies used in this workshop?

Several strategies were used to ensure rigor throughout the qualitative research process. Credibility was ascertained by recruiting participants from different disciplines and participating in five different workshop sessions. Observation notes were also used in order to confirm understanding of the content of the interviews. Coding tables were shared with other members of the project team. Dependability and confirmability were ascertained by the analysis process: a second investigator checked the reliability of coding; final themes were identified through consensus and were compared to original data in order to verify if they reflected an accurate representation of the participants’ experiences of the intervention.

Finally, a focus group was held with five of the seven workshop facilitators to discuss the strengths and weaknesses of the pedagogical process and identify how the workshop content and delivery could be improved or changed. This focus group lasted two hours. Notes were taken during this meeting (BD and BV) and a list of recommendations was drawn up and discussed with the project steering and pedagogical committees.

## Results

### Development of the intervention

#### Historical background to the project

In 2006, the Montérégie health and social services agency (ASSSM) adopted a strategic orientation to improve the quality of health services delivered in its region. One of the main strategies identified was that of engaging physicians in the continuous quality improvement process. It was considered essential to mobilize physicians because they play a central role in patient care management and share this responsibility with local healthcare managers. The DRMG was identified as an optimally positioned body for promoting critical reflection among physicians, facilitating the adoption and utilization of evidence-based guidelines, and developing quality improvement initiatives grounded in clinical practice needs. The other main strategy identified was that of increasing use of measurement tools that could provide direct feedback to the system. The Agency had recently developed expertise in extracting information from clinical-administrative databases using the ÉGIPSS model developed by Champagne et al. in 2005 [20]. However, the performance reports produced were mostly tailored to healthcare managers’ needs. The COMPAS project was launched to develop more relevant and practical performance reports adapted to clinicians’ specific needs and that would induce them to become involved in the continuous quality improvement process. The Agency’s wish was to innovate by linking top-down and bottom-up performance approaches. As pointed out by Ham [21], bottom-up changes introduced incrementally over time can result in more effective and enduring service improvements. This means building on evaluations of past and present improvements, fully engaging professionals in the process of change, and developing effective clinical leadership in the quest for performance improvements that benefit patients.

The project leaders ranked five chronic diseases in order of priority: diabetes, chronic obstructive pulmonary disease (COPD), heart diseases, depression, and cancer. Because of the prevalence of diabetes and the demonstrated effectiveness of interprofessional collaboration in improving diabetes management, this condition was chosen for the purpose of developing and piloting a first series of workshops.

#### Project governance

The COMPAS project is carried out with the input of four separate committees: a steering committee, coordination committee, scientific committee, and pedagogical committee. The steering committee’s role is to oversee the project and provide guidance for all decisions made regarding its orientation. It is composed of representatives of management and professional groups directly involved in the project: the head of the COMPAS project (MC), the head of the DRMG (LQ), the director of medical affairs at the ASSSM (JR), the project coordinator (BD), one representative of the Montérégie public health division (Direction de la santé publique at the Montérégie level), two representatives of the information and knowledge management division (Direction de la gestion de l’information et des connaissances), and three front-line clinicians (a physician, nurse, and pharmacist). The committee meets approximately three times a year to plan future activities and assess the project’s relevance and regional impact. The coordination committee is a sub-committee of the steering committee responsible for day-to-day decisions. It meets on a more regular basis to keep fuelling the project, organize workshop delivery, and coordinates the different committees’ work.

The scientific committee is an *ad hoc* committee convened to advise the steering committee on the choice of key indicators related to diabetes care and management that could be extracted from the clinical-administrative database, on the validation of the extraction process used, and on the interpretation of the results. It is composed of a research expert in chronic disease management, a research expert in pharmaco-epidemiology, an endocrinologist, a computer analyst with expertise in clinical-administrative data management, an expert in the field of health indicators and measures, and a general practitioner. The scientific committee met twice, reviewed the diabetes indicators selected by the steering committee, and made clear recommendations as to which indicators should be kept or added and how they should be interpreted.

Another *ad hoc* committee is also formed to develop the interprofessional educational intervention: the pedagogical committee. It is responsible for creating the workshop outline and content and for choosing the pedagogical strategies. This committee is chaired by two representatives of the continuing professional development (CPD) division of the FMOQ (CG and ML) because of their expertise in continuing education; it also includes key members of the steering committee.

One of the study investigators (BV) joined the pedagogical committee from the outset and was able to share her expertise on reflective learning to guide the intervention’s development. The pedagogical committee met four times. Internal validation (testing the intervention with the committee) and external validation (testing the intervention with a first group of clinicians) processes were performed to pre-test and improve the intervention. The pedagogical committee was also responsible for training the workshop facilitators.

### Description of the intervention

#### Theoretical basis of the intervention

The theoretical basis of the COMPAS project intervention reflected work motivation theory [22,23] and reflective learning [24]. One of the basic assumptions underlying the COMPAS intervention is that healthcare professionals are individuals who are absorbed in their everyday practice and who lack the time and opportunities to self-evaluate and self-monitor their practices [25]. However, in order to change and improve their practices, such as improving interprofessional collaboration and ensuring evidence-based diabetes management, these professionals need to recognize possible practice improvements and become involved in identifying strategies to achieve their shared ultimate goals, such as delivering high-quality health services and improving their patients’ health. Work motivation theory describes how to coach a person to instil a desire for continuous improvement [26]. In order to improve their practices, people are required to receive feedback that will allow them to assess whether or not they are achieving their pursued goals [27]. Feedback allows them to compare their own perceptions of their performance to external evaluation information, on which they can place more or less importance depending on the source’s perceived credibility and the informational value of the content. The aim of this comparison is to either confirm actual behaviour and goal attainment or identify gaps or dissonance between actual behaviour, pursued goals, and produced outcomes [28]. Dissonance is generated when, for example, clinicians believe they are giving appropriate and effective treatment but feedback indicates otherwise. Feedback is interpreted individually and in groups [29].

Reflection is the process whereby the individual and the group discuss and make sense of this information. In the reflective learning process, people focus first on the emotional response generated by the feedback and then make sense of the evaluative information received by drawing on their own experience and knowledge [24,30]. A second important step in the reflective learning process is that of being open to other perspectives and external knowledge (such as the content of clinical practice guidelines or the experience of another professional from another discipline) in order to reframe, when necessary, pursued goals and expected outcomes. Reflection serves the process of causal attribution [31]. The team may ascribe causes internally or externally and may perceive them as easily controllable or not. If the comparison between perceived actual practices and feedback validates the actual behaviour, the results of the reflective process will lead the team to make more effort and pay greater attention to achieving their shared goal [22,23]. However, if the comparison reveals dissonance, the results of the reflective process will lead to the identification of new cooperative goals requiring new strategies, greater efforts, and closer attention [23]. This will happen if the team perceives that it has a degree of control and if its members feel confident that they have the abilities to act on the situation.

The main outcome of the COMPAS project intervention is to achieve cooperative goal setting. Cooperative goal setting occurs when team members view the attainment of their respective goals as positively correlated and they work together for their mutual benefit [22,29]. Because the key factor differentiating a goal from a mere wish is whether or not people develop a plan to achieve it, action planning is also an important component of the intervention. By planning, individuals form an active mental representation of the target situation. Action planning [32] can help initiate action by specifying when, where, and how to act. People who develop action plans are more likely to act in the intended way [33], and they initiate the goal behaviour faster [34] than those who do not form action plans.

**Figure 1:**
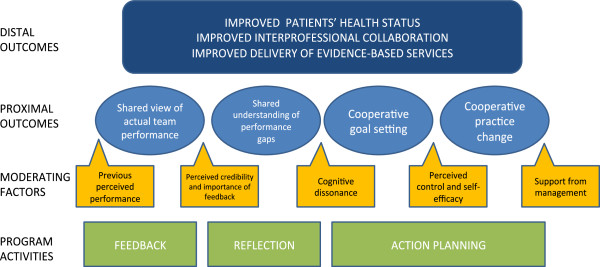
Program impact theory of the COMPAS project intervention.

As illustrated in Figure 1, the ultimate goal of the program impact theory underlying the COMPAS project intervention was found to be that of improving the health status of people with chronic diseases in Quebec’s Montérégie region. The two distal, or long-term, outcomes were identified as improved interprofessional collaboration and improved delivery of evidence-based services. Definition of the theoretical concepts included in the model is presented in Table 2. The proximal, or immediate, outcomes of the intervention were as follows: development of a shared view of actual team performance, achievement of a shared understanding of team performance gaps, setting of a cooperative and mutual goal, and adoption of cooperative practice changes. In accordance with work motivation theory [22,23], potential moderating factors that could facilitate or hinder the achievement of each proximal outcome were also included in the model: previous perceived performance, perceived credibility and amount of feedback, cognitive dissonance, perceived control and self-efficacy, and support from management for implementing practice change.

**Table 2:** Definition of the program theoretical concepts

**Theoretical concepts**	**Definitions**
*Proximal outcomes*	
Shared view of actual team performance	Using performance data information to achieve a mutual understanding of the strengths and weaknesses of chronic disease management in their region
Shared understanding of performance gaps	Achieving a mutual understanding of the disparities between the care delivered and the care they collaboratively wish to deliver
Cooperative goal setting	Translating the gap into a clear practice improvement goal and working together for the mutual benefit of improved chronic disease management
Cooperative practice change	Developing a team improvement strategy to achieve their mutual practice improvement goal
*Moderating factors*	
Previous perceived performance	If participants, before the workshop, have a positive or negative view of chronic disease management in their region
Perceived credibility and importance of feedback	If participants give value and credit to the information retrieved in the administrative database
Cognitive dissonance	If participants recognize a gap between actual and best practices
Perceived control and self-efficacy	If participants feel capable and confident they can improve chronic disease management and achieve their quality improvement goal
Support from management	If participants feel their organization and management will support the achievement of their quality improvement goal

#### Components of the intervention

The COMPAS intervention consists of a three-hour workshop led by two facilitators. It was designed to be offered to 20 to 25 professionals working in the same geographic area, thus serving the same population. The Montérégie region is divided into 11 sub-regions, some urban but most rural. It was decided that at least one workshop would be offered in each sub-region. Six workshop facilitators were trained to deliver the workshops in pairs: a physician and a pharmacist, or a physician and a nurse. Three physicians, two pharmacists, and one nurse received half a day of training. The main professional groups targeted by the diabetes workshops were physicians, nurses, and pharmacists, but other healthcare professionals were also invited to participate.

The COMPAS workshop is composed of three main activities: providing feedback, reflective learning, and action planning. The feedback intervention is delivered face-to-face at the beginning of the workshop. Performance indicators (Table 3) extracted from clinical-administrative databases (chosen by the steering committee and validated by the scientific committee) are presented to the participants by the workshop facilitators. The presentation takes about 40 minutes. It is concerned with past performance since the available databases date from 2006 and 2007. The feedback provided is neutral and does not contain any evaluative judgments. However, the presentation contains information from other sub-regions to allow comparisons between populations and service delivery and provides reminders of clinical practice guidelines (CPG) recommendations to support comparison with standards of care. A booklet summarizing Canadian diabetes guidelines for primary care practice is also included in the workshop material.

**Table 3:** List of indicators included in the feedback intervention

**Diabetic patients’ profile**	**Prescription profile**	**Health service use profile**
Number of diagnosed patients	Number of patients who are prescribed	Number of patients with an appointed family physician
Age	– anti-diabetic medication	Number of times patients consulted
Chronic disease score	– hypolipemic medication	– another GP
Frequency of diabetes complications	– anti-hypertensive	– cardiologist
– heart and vascular diseases	– medication	– internal medicine
– retinopathy	– AAS	– nephrologist
– kidney failure	– anti-diabetic medication	– eye specialist
	Adherence to medication regimen (valid prescription after 12 months and interruption of less than 14 days)	– community nurse
		– community nutritionist
		Frequency of GP visits
		Frequency of visits to a specialist
		Frequency of emergency visits
		Frequency of hospitalizations

The second component of the intervention is reflection. Reflection is an active process in which one witnesses one’s own experience in order to take a closer look at it and explore it in greater depth [25,30]. To support this reflective process, participants are asked at the start of the workshop to say what they perceive as the most important challenges they face in their practice in terms of improving diabetes management. These challenges are discussed in a large group. Following the feedback intervention, participants are asked to form small interprofessional groups of six to eight clinicians to make sense out of the information that was presented to them. The pedagogical committee developed a tool to guide their reflection process. Participants are asked to identify what surprises them with regard to patients’ characteristics, prescription profiles, and health services use profiles. They are then asked to identify what they perceive as the most important gaps between their actual team performance and what they consider a good team performance. In a large group, participants are invited to appraise their level of collaboration, discuss their respective and shared roles in diabetes management, and identify possible avenues for interprofessional practice improvement.

The third component of the intervention is action planning [35]. In small interprofessional groups, the participants are asked to select one priority practice improvement need that will become their shared goal. Small groups are invited to develop a plan that will allow them to achieve their goal. Using a template provided, they are asked to identify what they would do to achieve their goal, who would be in charge of the project, who would be doing what, and what the timeline would be, as well as to identify indicators that would help them measure whether they had achieved their goal. Action plans are presented on a poster board and discussed in a large group.

### Validation of the program impact theory

Five themes describing the workshop objectives emerged from the qualitative analysis of the interviews conducted with the workshop participants (Table 4). These five themes were the importance of: 1) adopting a regional perspective, 2) reflecting, 3) recognizing gaps between practice and guidelines, 4) collaborating, and 5) identifying possible practice improvements. During the interviews, most of the participants mentioned that one of the intervention’s aims was to allow them to form a regional vision of diabetes patients and diabetes service delivery.

“*This gives us an understanding of the follow-up done of diabetics and an overview of this follow-up for the region” (Participant #7).*

**Table 4:** Results of the thematic analysis in terms of categories and themes generated

**Research question**	**Themes**	**Categories**
According to you, what were the objectives of this workshop?	Adopting a regional perspective	Examining practices at a regional level
		Having a regional perspective of patient management
		Having a regional vision
	Reflecting	Reflecting on our own practice
		Reflecting on ways by which we can improve our practice
		Understanding how we manage our case load
		Allowing us to reflect on quality of care improvement
		Reflecting on follow-up of patients
		Analyzing the global situation
		Recognizing our strengths and weaknesses
	Recognizing gaps between practice and guidelines	Comparing practice with clinical guidelines
		Comparing with good practices
		Recognizing gaps between practices and
		guidelines
		Recognizing the relevance of performance feedback
	Collaborating	Working in interdisciplinary
		Working in collaboration
		Having a better vision of each other roles
		Being more aware of what other professionals are doing
		Changing our view of collaboration
		Recognizing complementarities between professionals
		Facilitating networking
		Knowing what is done elsewhere in order to improve our practice
	Identifying possible practice improvements	Identifying one aspect of practice we wish to improve
		Identifying possible ways to improve practice
		Offering better care
		Providing more homogenous and standardized chronic disease care in the region
		Improving treatment adherence
		Developing clinical tools

They saw the objective of presenting data from the clinical-administrative database as that of giving them a broader perspective and allowing them to compare their practices to those in other regions. This comparison was seen as useful, for example, for understanding how the availability of specialized services such as ophthalmology impacted on retinopathy screening.

Reflection was also an objective perceived by participants. They said it was helpful for recognizing the team’s strengths and weaknesses and gaining a better understanding of how patient follow-up is performed. One participant put it this way:

“The reflective approach is not the usual approach we are accustomed to in workshops. It means doing our own reflection. We weren’t given data just for the sake of it, but so that we could interpret our profile ourselves” (Participant #5).

The interviewees also commented that the intervention was useful for identifying gaps between actual practice and clinical practice guidelines. This helped increase their awareness of the fact that even if they were knowledgeable about the guidelines, they did not always apply them in their daily practices.

“It makes us think about the follow-up we do. We are more careful afterwards about following the guidelines” (Participant #5).

“The objective was not to question the guidelines, but rather to help us see whether we applied them” (Participant #8).

Collaborating was the objective most frequently mentioned by the participants. They said that the workshop increased their understanding of other professionals’ roles and their complementarity, changed their previous assumptions about possible collaborations, and facilitated networking among them. For the participating nurses and physicians, the main outcome of the workshop was new possible collaborations with pharmacists to improve diabetes management.

“Pharmacists are invaluable collaborators who should be better integrated into the different steps involved in care” (Participant #9).

“It changed my view of pharmacists. I didn’t think they could have such an influence” (Participant #6).

“We pharmacists know that the other professionals have a hard time recognizing that we see patients 12 times a year and know a lot about clients’ adherence [to treatment regimens]. We are poorly understood” (Participant #8).

The fifth theme was the importance of “identifying possible practice improvements.” Participants mentioned that the workshop allowed them to target what they wished to improve in their collaborative management of diabetes in order to de-clutter and reorganize services to improve the healthcare system, deliver more homogenous and standardized care in the region, increase treatment adherence, and develop new tools to improve follow-up and interprofessional collaboration.

“We targeted an action that would improve screening for retinopathy, and it has already been implemented in my clinic” (Participant #2).

### Feasibility and acceptability of the intervention

The task of delivering the workshops was more challenging than expected. In the regions, medical education is rarely offered to mixed professional groups, and busy physicians are responsible for organizing the CE activity calendar for peers. Even though an advertisement for the intervention was published in various local and provincial newspapers, word-of-mouth proved to be the most effective way of convincing one CME representative to organize a workshop. Inviting community pharmacists and nurses to the workshops was perceived as time-consuming and as an unusual modus operandi. Thus, at some workshop sessions, there was a good balance between the number of physicians, nurses, and pharmacists, while at others, there were insufficient nurses and pharmacists for them to participate in all the small group discussions. We also observed that when there were prior habits of collaborating or when nurses and physicians were already sharing responsibilities for the management of diabetic patients, it facilitated the reflective and action-planning processes. When a pattern of collaboration was not already established, the workshop’s aim was limited to having the participants get to know one another and explore their respective roles and possible collaborations. Three hours was sufficient to deliver the three workshop components. However, some participants mentioned that less time should be devoted to the feedback intervention and more time allowed for reflection on interprofessional collaboration and action plan development. The pedagogical material that had been developed to support small group work was found to be cumbersome. Participants were asked to use large sheets of paper to write down the outcomes of their reflection and their action plan and then to stick these sheets of paper onto a large board. However, limited room space and unclear or small handwriting limited the usefulness of these materials for purposes of the large group discussion.

The workshop’s acceptability was found to be very good. Even if the feedback/reflective-learning approach represents a new way of delivering CE activities in Quebec, most of the participants enjoyed their experience. Satisfaction with the workshop content and material was quite high (Table 5). However, our observation of the workshops and the participant satisfaction reports also showed that, for each workshop, a few participants appeared to prefer more traditional CME activities, considered that they had not learned much about diabetes care, and had trouble identifying how this intervention would be useful for improving their practices. Participants appreciated the information presented in the feedback intervention, despite being made aware of the fact that the validity of data extracted from clinical-administrative databases is limited since these data are dependent on codes used for health service billing purposes. Importance appeared to be placed on the fact that the data were endorsed by the Montérégie health agency and the DRMG. Nevertheless, in two of the observed workshop sessions, a physician challenged the information presented in front of the group. In these cases, the workshop facilitators were familiar with the information presented and it was very important that they took the time to clarify the situation when misunderstandings arose. When the facilitators were unsure of the facts or unable to reassure participants, this may have had an impact on the small group discussions, especially in the group of skeptical participants.

**Table 5:** Mean satisfaction scores* reported by participants (n = 86)

**Items**	**Mean**	**SD**
The goals of the workshop matched my needs.	3.41	0.15
There was sufficient interaction between the facilitator and participants.	3.67	0.34
This workshop will have an impact on my practices.	3.41	0.26

*Satisfaction score: 4 = very satisfied, 3 = quite satisfied, 2 = somewhat satisfied, 1 = not satisfied.

Following the delivery of a first series of workshops on diabetes, four facilitators out of six were still able to and very interested in continuing to participate in the COMPAS project. One physician and one nurse had to withdraw because of work- and family-schedule conflicts. The focus group with the workshop facilitators and the project coordinator generated a list of challenges encountered during workshop delivery and of perceived possible improvements. The difficulties cited pertained to workshop planning and the need to use more formal strategies to reach more physicians and nurses working in primary care settings, as well as community pharmacists. The need to improve the ways in which the facilitators support small group reflection and action planning was also seen as important. The use of pencils and paper was considered an impractical and outdated means of sharing information with the large group. Also, the facilitators mentioned that they would appreciate having the opportunity to present previous action plans developed by other teams in other regions to stimulate small team work in instances where teams experience difficulty in finding a cooperative goal and drawing up an action plan. The facilitators perceived workshop follow-up as a very important means of encouraging action plan implementation and team practice change. It was decided that the project coordinator would do a one-, three-, and six-month follow-up with the workshop participants by e-mail.

## Discussion and conclusion

The aim of this paper was to describe the theory underlying the COMPAS intervention delivered to primary care professionals for the purpose of improving interprofessional collaboration and their involvement in improvement of the quality of chronic disease care. As described in a literature review performed by Xyrichis [36], several barriers exist that hinder interprofessional collaboration in primary care. Some of these barriers are lack of communication between team members, lack of a clear understanding of each other’s professional roles and responsibilities, and lack of practice evaluation. Recommendations have been made to increase the number of team meetings, identify team goals, and conduct team audits. The intervention developed in the Montérégie region addressed these barriers by bringing together professionals from numerous disciplines who are involved in chronic disease management, providing them with feedback on their practices and performance, and having them develop their own action plans to improve the quality of their services. As pointed out by Ham [21], clinicians need time and space to review established practices and to introduce new and more effective ways of delivering services. It is also important that the interventions carried out to improve clinicians’ involvement be based on what motivates them in their daily work.

The theoretical basis of the COMPAS project intervention reflected work motivation theory [22,23] and reflective learning [24]. Providing feedback and opportunities for reflective learning are two recommended strategies for continuing professional development, and both have demonstrated their effectiveness in supporting the implementation of practice changes [14,25,37]. However, the research evidence is still unclear as to how we should deliver these interventions. Combining both into a three-hour workshop session was a challenge. Right from the beginning of our study, it appeared important to the research team that the developers of the COMPAS intervention should gain a clear understanding of the intervention’s expected outcomes and its action mechanisms before proceeding to its evaluation [17]. In research, most evaluation involves a “black box” approach: the inner mechanisms of the intervention are not explicit or are unknown [17]. The value of conducting theory-driven evaluations is that they have the potential to contribute to an evolving understanding of the nature of the change processes that programs bring about and of the ways in which these processes can be optimized [17,18]. This approach to evaluation is enriching since it provides evaluators with a thorough understanding of the most effective program components, the mediating processes through which they work, and the moderating factors related to participants and the context influencing the achievement of expected outcomes. The development of the program impact theory underlying the COMPAS project intervention will improve our ability to measure the intervention’s real impacts on practice change and further elucidate the factors influencing the intervention.

Our observation of the workshop sessions and the interviews conducted with the participants confirmed that the objectives of the COMPAS team’s intervention indeed targeted the improvement of interprofessional collaboration and quality of care. Participants described the participating physicians’ and nurses’ lack of awareness of how pharmacists could contribute to chronic disease management in primary care. Pharmacists’ practices are often perceived as being based on physicians’ prescriptive decisions and on technical aspects of dispensing medication [38]. However, the small group discussions increased the participants’ awareness of the active role that pharmacists can play in diabetes management and in providing continuity of care since they see patients regularly and are well-placed to know whether patients are adhering to their medication and treatment plans. They can educate clients on a regular basis and offer self-management support by answering questions and referring patients to appropriate professionals. Another important objective of the intervention was to help participants recognize practice gaps and to create opportunities for them to become personally involved in the improvement of service delivery. The way in which the feedback intervention was delivered promoted the development of a community-oriented primary care (COPC) vision [39,40]. Community-oriented primary care is about providing accessible, comprehensive, coordinated, continuous, and accountable healthcare services to a defined community. Community refers to a geographic group for which a health organization is responsible. The form of feedback delivered in the COMPAS project intervention facilitates the process of community-oriented primary care delivery because it allows professionals to improve their knowledge of the characteristics of the community they serve and of the prevalence of specific health problems in that community, but also to improve healthcare services, address community needs, and monitor the effectiveness of their interventions [41]. COPC has been described as a promising approach for health promotion and disease prevention and would appear highly conducive to improving integrated care for chronic disease management [40,41].

One limitation of this study is the small number of professionals recruited for the interviews. Busy clinicians lack time to participate in research projects, especially projects that do not directly involve patients. The professionals volunteered to participate to the interviews and were maybe more satisfied or motivated than other workshop participants. Nevertheless, participants’ answers were converging and most of the themes were identified after a few interviews. The project coordinator (BD) conducted the interviews and the qualitative analysis. An interview guide was used to minimize the influence of his personal interpretation. His knowledge of the intervention was however helpful to help participants talk about their learning experience. In order to increase credibility, a second investigator (BV) was involved at every step of the analysis process. Triangulation was also used: results from the interviews were compared to the workshop observation notes and the participants’ satisfaction evaluation.

However, this study was limited to surfacing and describing the program impact theory underlying an innovative, interprofessional continuing education program. It does not confirm its effectiveness and its evaluation will require the use of an appropriate experimental research design. Program theory development increases our understanding of the expected mechanisms of action of the intervention but do not confirm its real outcomes and if it did lead to practice changes. However, these preliminary results have shown that the intervention does indeed target its expected outcomes. Our hypothesis is that a three-hour workshop alone cannot lead to major changes in practice. Long-term interventions are needed to support this complex change process. That said, using feedback and reflective learning increases participants’ awareness of practice gaps and the need to improve how they collaborate. To maximize the intervention’s impact, a follow-up intervention will be carried out within one year of the initial workshop to support implementation of the developed action plans. The project team is also confident that the COMPAS intervention will have an impact on practice if it is repeated and if these types of reflective learning experiences and team meetings are integrated into practice on at least an annual basis. Recently we launched a new series of workshops for chronic obstructive pulmonary diseases (COPD), and it is our hope that professionals will become more and more familiar with the COMPAS approach in order to increase their involvement in the quality improvement of health services in the communities they serve.

## Competing interests

The authors declare that they have no competing interests.

## Authors’ contributions

BV, JG, MC and JR conceived and designed the study. MC, JR, LQ, CG, ML, JF contributed to the development of the intervention. JR, MC, LQ, BV and BD participated in the coordination and the implantation of the interventions. JF supervised all the databases extractions. BV and BD did the data collection and wrote the original manuscript. All authors read and approved the manuscript.

## Pre-publication history

The pre-publication history for this paper can be accessed here:

http://www.biomedcentral.com/1472-6963/13/106/prepub
